# Thyroid hormone actions are temperature-specific and regulate thermal acclimation in zebrafish (*Danio rerio*)

**DOI:** 10.1186/1741-7007-11-26

**Published:** 2013-03-26

**Authors:** Alexander G Little, Tatsuya Kunisue, Kurunthachalam Kannan, Frank Seebacher

**Affiliations:** 1 School of Biological Sciences, A08 University of Sydney, Science Road, Sydney, NSW, 2006, Australia; 2 School of Public Health, Wadsworth Center, New York State Department of Health, Albany, NY, 12201-0509, USA; 3 State Key Laboratory of Urban Water Resources and Environment, IJRC PTS, Harbin Institute of Technology, Harbin, 150090, China

**Keywords:** Thyroid hormone, Zebrafish, Temperature, Cold acclimation, Hypothyroid, Ectotherm, Metabolism, Thermal plasticity, Thermal response

## Abstract

**Background:**

Thyroid hormone (TH) is best known for its role in development in animals, and for its control of metabolic heat production (thermogenesis) during cold acclimation in mammals. It is unknown whether the regulatory role of TH in thermogenesis is derived in mammals, or whether TH also mediates thermal responses in earlier vertebrates. Ectothermic vertebrates show complex responses to temperature variation, but the mechanisms mediating these are poorly understood. The molecular mechanisms underpinning TH action are very similar across vertebrates, suggesting that TH may also regulate thermal responses in ectotherms. We therefore aimed to determine whether TH regulates thermal acclimation in the zebrafish (*Danio rerio*). We induced hypothyroidism, followed by supplementation with 3,5-diiodothyronine (T_2_) or 3,5,3^′^-triiodothyronine (T_3_) in zebrafish exposed to different chronic temperatures. We measured whole-animal responses (swimming performance and metabolic rates), tissue-specific regulatory enzyme activities, gene expression, and free levels of T_2_ and T_3_.

**Results:**

We found that both T_3_ and the lesser-known T_2_, regulate thermal acclimation in an ectotherm. To our knowledge, this is the first such study to show this. Hypothyroid treatment impaired performance measures in cold-acclimated but not warm-acclimated individuals, whereas supplementation with both TH metabolites restored performance. TH could either induce or repress responses, depending on the actual temperature and thermal history of the animal.

**Conclusions:**

The low sensitivity to TH at warm temperatures could mean that increasing temperatures (that is, global warming) will reduce the capacity of animals to regulate their physiologies to match demands. We suggest that the properties that underlie the role of TH in thermal acclimation (temperature sensitivity and metabolic control) may have predisposed this hormone for a regulatory role in the evolution of endothermy.

## Background

During thermal acclimation, ectotherms can shift their reaction norms by modifying the thermal sensitivities of their metabolic and other physiological pathways. This process can be mediated by changes in enzyme concentrations and mitochondrial biogenesis, modification of cell and mitochondrial membranes, and conformational changes that optimize enzyme efficiencies at different temperatures [1-3]. Despite the importance of acclimation for physiology, ecology, and conservation, the overarching mechanisms governing this process in ectotherms remain unknown [3]. We hypothesize that thyroid hormone (TH) regulates the thermal-acclimation response. Testing this hypothesis is important to understand how animals respond to temperature change. It is also important in an evolutionary context, because the ancestral function(s) of TH may have predisposed it for its central regulatory role in the evolution of endothermy.

TH has garnered much attention for its roles in metabolic heat production (thermogenesis) and energy expenditure [4-8] in mammals, which could be exploited for the treatment of diseases including obesity, type 2 diabetes, and metabolic syndromes [9-15], but the complexity of the TH system is far from resolved. Several TH metabolites can stimulate physiological responses through a wide range of signaling pathways that are subject to many levels of biological regulation [16-21]. In vertebrates, TH is produced in the thyroid gland primarily as thyroxine (T_4_), and is metabolized to 3,5,3^′^-triiodothyronine (T_3_) and 3,5-diiodothyronine (T_2_) by deiodinase enzymes (D1, D2 and D3) in peripheral tissues [22]. Other TH isomers exist, but are either physiologically inactive or have very low activity [23]. T_3_ was originally believed to be the only physiologically active TH because of its unique affinity for TH receptors, which regulate the expression of target genes transcriptionally by binding to thyroid response elements (TREs) in their promoters [23]. Recently, however, T_2_ was also found to stimulate metabolism, but through non-genomic (post-transcriptional) pathways, which are as yet poorly understood [23]. T_2_ acts at different cellular levels (including plasma membrane, cytosol, and mitochondria) and elicits much quicker responses than T_3_[18,23]. Although T_2_ has been shown to stimulate metabolism, its physiological relevance is still in question [23].

TH is ubiquitous across all vertebrates [4,24], and even stimulates growth and development in many invertebrate groups [25-32]. Interestingly, TH can produce drastically different responses in different animal groups [24,33]. Generally, TH regulates growth and development in vertebrates and invertebrates, but additionally regulates metabolism and thermogenesis during cold exposure in mammals. However, these functionally distinct roles are underpinned by overlapping physiological and biochemical pathways [33]. Energy metabolism and its control are highly conserved in vertebrates [34], but it is unknown whether the role of TH in mediating thermal responses is independently derived in mammals, or whether it is also present in earlier vertebrates. In all animals, biochemical pathways are sensitive to acute changes in temperature. However, endotherms and many ectotherms regulate, or acclimate, their metabolism to compensate for longer-term (days to weeks) thermal variation in their environments. We hypothesize that, as in endotherms, TH regulates these physiological responses of ectotherms to chronic changes in their thermal environment.

Specifically, we assessed the metabolic role of TH during thermal acclimation in the zebrafish (*Danio rerio*). The zebrafish was chosen as a model because fish occupy an early position in vertebrate evolution, and the zebrafish in particular has become an important biomedical model for thyroid-related disease, including obesity, cardiovascular disease, and diabetes [35,36]. We used a multi-factorial experimental design in which we induced hypothyroidism, followed by supplementation with T_2_ and T_3_ (plus normal thyroid controls) in zebrafish exposed to different chronic and acute temperature combinations. We measured whole-animal responses (swim performance, metabolic rates, and metabolic scope), and determined tissue-specific protein function (activities of regulatory enzymes), metabolic gene expression (liver and muscle), and levels of free T_2_ and T_3_ to determine whether TH is the mechanism that drives thermal acclimation in ectotherms. We hypothesized that hypothyroidism would impair acclimation responses, and that supplementation with T_3_ and T_2_ would restore acclimation in hypothyroid fish.

## Results

### Thyroid hormone levels

Levels of both T_3_ and T_2_ were lower in muscle tissue from cold-acclimated fish (Table 1). The TH levels in cold-acclimated hypothyroid fish supplemented with T_3_ and T_2_ verified that our hypothyroid and supplementation treatments were effective (Table 2).

**Table 1:** **Muscle-specific levels of T**_**2 **_**and T**_**3 **_**in cold-acclimated and warm-acclimated normal thyroid zebrafish**

	**Cold-acclimated**		**Warm-acclimated**	
**Sample**	**T**_**2**_**, ng/g**	**T**_**3**_**, ng/g**	**T**_**2**_**, ng/g**	**T**_**3**_**, ng/g**
1	2.45	3.35	3.11	6.23
2	<1.00	<1.00	1.25	9.57
3	1.91	2.83	1.56	7.05
4	4.27	7.00	2.96	28.70
5	1.40	1.95	1.70	39.10
6	<1.00	1.54	5.25	531.00
Mean	1.71 to 2.00	2.80 to 2.95	2.64	103.61
SEM	0.21	0.36	0.25	34.97

**Table 2:** **Muscle-specific levels of T**_**2 **_**and T**_**3 **_**in cold-acclimated hypothyroid zebrafish supplemented with T**_**2 **_**or T**_**3**_

	**T**_**2**_**-supplemented**		**T**_**3**_**-supplemented**	
**Sample**	**T**_**2**_**, ng/g**	**T**_**3**_**, ng/g**	**T**_**2**_**, ng/g**	**T**_**3**_**, ng/g**
1	2.66	0.00	0.00	107.00
2	<5.00	0.00	0.00	40.80
3	1.73	0.00	0.00	52.90
4	<5.00	0.00	0.00	30.60
5	<5.00	0.00	0.00	40.00
6	<5.00	0.00	0.00	63.10
7	56.50	<1.00	0.00	147.00
8	52.10	1.97	0.00	77.00
9	44.00	1.75	NA	NA
Mean	17.50 to 19.70	0.42 to 0.52	0.00	69.80
SEM	2.80	0.09	0.00	4.95

Abbreviations: NA, not applicable.

### Effects of cold acclimation

**Figure 1:**
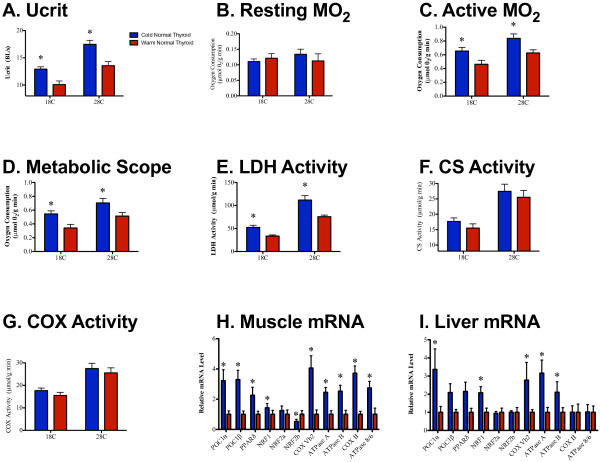
**Thermal-acclimation responses in normal thyroid fish.** (**A**) U_crit_, (**B**) resting metabolic rate, (**C**) active metabolic rate, (**D**) metabolic scope, (**E**) maximal lactate dehydrogenase (LDH) activity (**F**), maximal citrate synthase (CS) activity, (**G**) maximal cytochrome c oxidase (COX) activity, (**H**) muscle mRNA transcript levels and (**I**) liver mRNA transcript levels in cold-acclimated and warm-acclimated normal thyroid fish (blue and red, respectively); * Significant difference between acclimation treatments.

Cold acclimation significantly increased sustained swimming performance (critical sustained swim speed; U_crit_) at acute test temperatures of both 18°C and 28°C (Figure 1A; Table 3; see Additional file 1: Table S1). Cold-acclimated fish (kept for 3 weeks at 18°C) compensated for the limiting effect of low temperature, and swam as well at the 18°C acute test temperature as warm-acclimated (3 weeks at 28°C) fish did at a test temperature of 28°C.

There was no effect of acclimation temperature on resting metabolic rate, but cold acclimation significantly increased active metabolic rate and, hence, metabolic scope (Figure 1B–D; Table 3). There was a significant increase in maximal lactate dehydrogenase (LDH) activity in the cold-acclimated fish, but no effect of acclimation treatment on maximal citrate synthase (CS) or cytochrome c oxidase (COX) activities (Figure 1E–G; Table 3; see Additional file 1: Table S1). Cold-acclimation significantly increased transcript levels of the transcriptional coactivators PGC1α and PGC1β, their target transcription factors PPARδ, NRF1, and NRF2b, subunits of the mitochondrial enzymes COX (COX VB2 and COX II), and F_o_F_1_-ATPase (ATPase A, ATPase B, and ATPase 8/6) in muscle, and PGC1α, NRF1, NRF2b, COX VB2, ATPase A, and ATPase B in liver (Figure 1H,I; Table 4; Table 5; see Additional file 1: Table S1).

**Table 3:** Three-way permutational multivariate analysis of variance (PERMANOVA) results comparing parameters between cold-acclimated and warm-acclimated normal thyroid and hypothyroid fish

	**AT**^**a**^	**H**^**b**^	**TT**^**c**^	**AT**^**a **^**× H**^**b**^	**AT**^**a **^**× TT**^**c**^	**H**^**b **^**× TT**^**c**^	**AT**^**a **^**× H**^**b **^**× TT**^**c**^
U_crit_ ^d^							
*F*	5.319	12.900	14.768	5.711	0.0088	2.079	0.838
d.f	1, 69	1, 69	1, 69	1, 69	1, 69	1, 69	1, 69
*P*	0.014*	<0.001**	<0.001**	0.017*	0.883	0.121	0.351
RMR							
*F*	3.767	1.216	3.069	1.153	0.427	4.479	2.411
d.f	1, 59	1, 59	1, 59	1, 59	1, 59	1, 59	1, 59
*P*	0.031*	0.273	0.048	0.297	0.653	0.032*	0.103
AMR							
*F*	12.790	8.354	12.430	0.567	1.064	0.112	0.200
d.f	1, 59	1, 59	1, 59	1, 59	1, 59	1, 59	1, 59
*P*	<0.001**	0.004**	<0.001**	0.471	0.305	0.829	0.717
MS							
*F*	8.488	9.140	8.251	1.280	0.772	0.352	1.031
d.f	1, 59	1, 59	1, 59	1, 59	1, 59	1, 59	1, 59
*P*	0.004**	0.004**	0.007**	0.247	0.371	0.637	0.293
LDH^e^							
*F*	11.547	0.642	145.460	5.611	3.886	0.291	2.346
d.f	1, 72	1, 72	1, 72	1, 72	1, 72	1, 72	1, 72
*P*	<0.001**	0.445	<0.001**	0.018*	0.045*	0.645	0.107
CS^e^							
*F*	0.505	0.010	45.700	2.410	0.040	0.006	0.246
d.f	1, 72	1, 72	1, 72	1, 72	1, 72	1, 72	1, 72
*P*	0.497	0.991	<0.001**	0.122	0.935	0.992	0.674
COX^e^							
*F*	1.920	2.122	16.849	3.872	1.027	0.361	0.406
d.f	1, 56	1, 56	1, 56	1, 56	1, 56	1, 56	1, 56
*P*	0.159	0.148	<0.001**	0.044*	0.314	0.551	0.548

Abbreviations: AMR, active metabolic rate, CS, citrate synthase; COX, cytochrome oxidase; MS, metabolic scope; LDH, lactate dehydrogenase; RMR, resting metabolic rate.

^a^Acclimation temperature.

^b^Thyroid treatment.

^c^Test temperature.

^d^Swimming performance.

^**e**^Maximal activity.

*P<0.05.

**P<0.01.

**Table 4:** Two-way permutational multivariate analysis of variance (PERMANOVA) results comparing muscle mRNA levels between cold-acclimated and warm-acclimated normal thyroid and hypothyroid fish

	**Acclimation temperature**			**Thyroid status**			**Acclimation temperature × Thyroid Status**		
**Gene**	***F***	**d.f.**	***P***	***F***	**d.f.**	***P***	***F***	**d.f.**	***P***
PGC1α	3.330	1, 34	0.041*	6.454	1, 34	0.001**	4.357	1, 34	0.026*
PGC1β	12.690	1, 34	0.001**	2.677	1, 34	0.09	2.068	1, 34	0.108
PPARδ	5.708	1, 31	0.010*	0.172	1, 31	0.827	2.511	1, 31	0.117
NRF1	5.069	1, 28	0.021*	0.921	1, 28	0.343	0.268	1, 28	0.714
NRF2a	1.434	1, 28	0.239	0.145	1, 28	0.885	1.086	1, 28	0.302
NRF2b	4.108	1, 17	0.045*	0.557	1, 17	0.537	0.060	1, 17	0.948
COX Vb2	1.996	1, 33	0.124	3.786	1, 33	0.029*	7.293	1, 33	0.002**
ATPase A	3.612	1, 27	0.048*	2.260	1, 27	0.099	3.136	1, 27	0.043*
ATPase B	1.948	1, 27	0.148	1.216	1, 27	0.269	5.903	1, 27	0.012*
COX II	12.410	1, 25	0.001**	3.850	1, 25	0.026*	2.478	1, 25	0.079
ATP 8/6	7.221	1, 24	0.004**	8.229	1, 24	0.002**	2.631	1, 24	0.068

Abbreviations: ATPase A, F_0_F_1_-ATPase subunit A; ATPase B, F_0_F_1_-ATPase subunit B; ATPase 8/6, F_0_F_1_-ATPase subunits 8 and 6; COX II, Cytochrome c oxidase subunit 2; COX Vb2, cytochrome c oxidase subunit 5b2; d.f. degrees of freedom; NRF1, Nuclear respiratory factor 1; NRF2a, Nuclear respiratory factor 2a; NRF2b, Nuclear respiratory factor 2b; PGC1α, Peroxisome proliferator-activated receptor γ coactivator 1-α; PGC1β, Peroxisome proliferator-activated receptor γ coactivator 1-β; PPARδ, Peroxisome proliferator-activated receptor δa and δb.

*P<0.05.

**P<0.01.

**Table 5:** Two-way permutational multivariate analysis of variance (PERMANOVA) results comparing liver mRNA levels between cold-acclimated and warm-acclimated normal thyroid and hypothyroid fish

	**Acclimation temperature**			**Thyroid status**			**Acclimation temperature × thyroid status**		
**Gene**	***F***	**d.f.**	***P***	***F***	**d.f.**	***P***	***F***	**d.f.**	***P***
PGC1α	13.672	1, 32	0.001**	2.597	1, 32	0.095	1.521	1, 32	0.238
PGC1β	26.692	1, 29	0.001**	1.191	1, 29	0.274	6.374	1, 29	0.011*
PPARδ	25.657	1, 31	0.001**	1.128	1, 31	0.324	6.139	1, 31	0.010*
NRF1	32.817	1, 26	0.001**	0.784	1, 26	0.400	3.464	1, 26	0.050*
NRF2a	7.869	1, 22	0.004**	1.163	1, 22	0.290	5.015	1, 22	0.020*
NRF2b	15.085	1, 24	0.001**	0.578	1, 24	0.497	6.708	1, 24	0.017*
COX Vb2	9.789	1, 20	0.005**	3.490	1, 20	0.049*	0.336	1, 20	0.672
ATPase A	23.119	1, 32	0.001**	0.292	1, 32	0.713	1.109	1, 32	0.308
ATPase B	6.456	1, 33	0.007**	0.097	1, 33	0.942	1.604	1, 33	0.189
COX II	6.458	1, 22	0.007**	1.240	1, 22	0.295	6.377	1, 22	0.013*
ATP 8/6	4.056	1, 21	0.06	0.422	1, 21	0.571	3.904	1, 21	0.044*

Abbreviations: ATPase A, F_0_F_1_-ATPase subunit A; ATPase B, F_0_F_1_-ATPase subunit B; ATPase 8/6, F_0_F_1_-ATPase subunits 8 and 6; COX II, Cytochrome c oxidase subunit 2; COX Vb2, Cytochrome c oxidase subunit 5b2; d.f. degrees of freedom; NRF1, Nuclear respiratory factor 1; NRF2a, Nuclear respiratory factor 2a; NRF2b, Nuclear respiratory factor 2b; PGC1α, Peroxisome proliferator-activated receptor γ coactivator 1-α; PGC1β, Peroxisome proliferator-activated receptor γ coactivator 1-β; PPARδ, Peroxisome proliferator-activated receptor δa and δb.

*P<0.05.

**P<0.01.

### The effects of hypothyroid treatment on responses that acclimated

**Figure 2:**
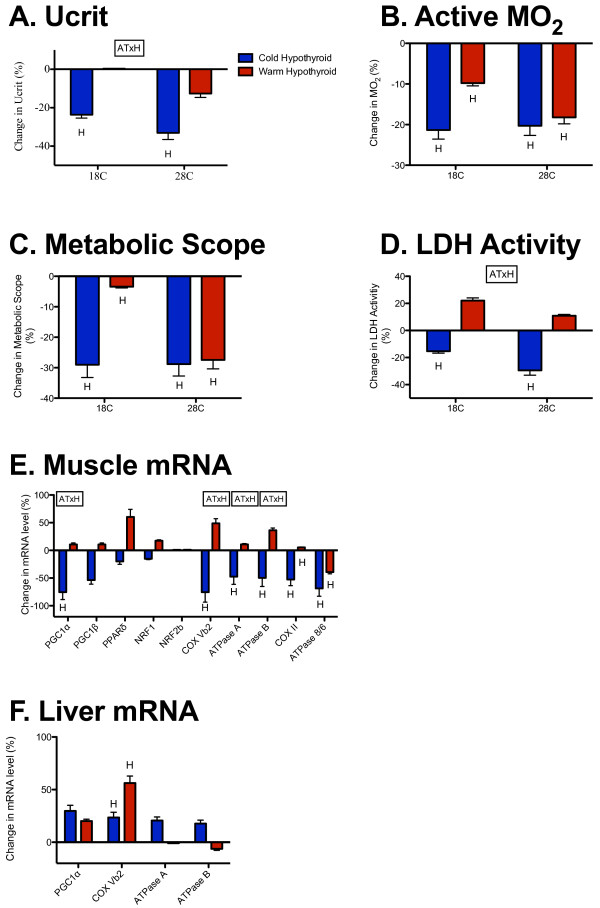
**Effects of hypothyroid treatment on cold-acclimation responses.** Percentage difference in (**A**) U_crit_, (**B**) active metabolic rate, (**C**) metabolic scope, (**D**) maximal lactate dehydrogenase (LDH) activity, (**E**) muscle mRNA transcript levels and (**F**) liver mRNA transcript levels in cold-acclimated and warm-acclimated hypothyroid fish (blue and red, respectively) relative to normal thyroid fish. H, significant effect of hypothyroid treatment; AT × H, significant interaction between acclimation temperature and hypothyroid treatment.

Hypothyroid treatment significantly decreased the sustained swim speed in the cold-acclimated fish, but it had no effect on swimming performance in the warm-acclimated fish (Figure 2A; Table 3; see Additional file 1: Table S1). Hypothyroid treatment significantly decreased active metabolic rate and metabolic scope at both acclimation temperatures (Figure 2B,C; Table 3). Hypothyroid treatment significantly decreased LDH activity in muscle of cold-acclimated fish but it had no effect on warm-acclimated fish (Figure 2D; Table 3; see Additional file 1: Table S1). There was also a significant interaction between acclimation treatment and test temperature on the activity of LDH.

Paralleling responses of swimming performance and LDH activity, there were significant interactions between hypothyroid treatment and acclimation temperature for transcript levels of PGC1α, COX VB2, COX II, ATPase A, ATPase B, and ATPase 8/6 in muscle, whereby hypothyroid treatment significantly decreased transcript levels in cold-acclimated fish but had no effect on warm-acclimated fish (Figure 2E; Table 4; see Additional file 1: Table S1). There were significant effects of hypothyroid treatment on COX II and ATPase 8/6 in muscle and COXVB2 in liver (Figure 2E,F; Table 4; Table 5). Hypothyroid treatment had no significant effect on transcript levels of PGC1β, PPARδ, NRF1, and NRF2b in muscle, or on PGC1α, ATPase A, and ATPase B in liver (Figure 2E,F; Table 4; Table 5). For those genes that responded to acclimation treatments, there was a tendency for hypothyroid treatment to decrease transcript levels in cold-acclimated fish and increase transcript levels in warm-acclimated fish.

### The effects of hypothyroid treatment on response measures that did not acclimate

**Figure 3:**
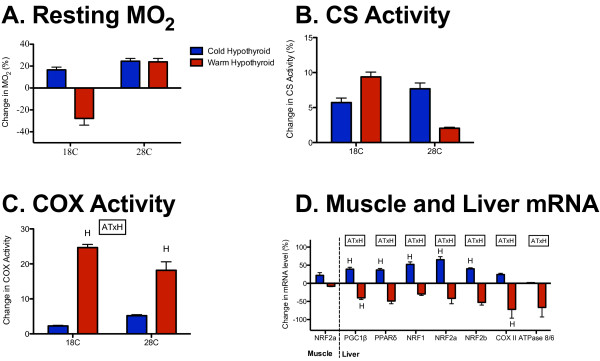
**Effects of hypothyroid treatment on measures that do not acclimate to cold.** Percentage difference in (**A**) resting metabolic rate, (**B**) maximal citrate synthase (CS) activity, (**C**) maximal cytochrome c oxidase (COX) activity, and (**D**) muscle and liver mRNA transcript levels in cold-acclimated and warm-acclimated hypothyroid fish (blue and red, respectively) relative to normal thyroid fish; H. significant effect of hypothyroid treatment; AT × H, significant interaction between acclimation temperature and hypothyroid treatment.

Hypothyroidism decreased resting metabolic rate at the 18°C test temperature, but increased resting metabolic rate at the 28°C test temperature (Figure 3A; Table 3). Hypothyroid treatment had no significant effect on CS activity or muscle transcript levels for NRF2a (Figure 3B,D; Table 3; Table 4). There was a significant interaction between hypothyroid treatment and maximal COX activity, whereby hypothyroidism significantly increased COX activity in muscle of the warm-acclimated fish but had no effect on the cold-acclimated fish (Figure 3c; see Table 3, Additional file 1: Table S1).

There were significant interactions between hypothyroid treatment and acclimation temperature for liver transcript levels of PGC1β, PPARδ, NRF1, NRF2a, NRF2b, COX VB2, COX II, and ATPase 8/6, whereby hypothyroidism significantly increased the transcript levels of PGC1β, PPARδ, NRF1, NRF2a, and NRF2b in cold-acclimated fish and significantly reduced transcript levels of PGC1β and COX II in warm-acclimated fish (Figure 3D; Table 5; see Additional file 1: Table S1). Overall, for those genes that did not respond to acclimation treatments, there was a tendency for hypothyroidism to increase transcript levels in cold-acclimated fish and decrease transcript levels in warm-acclimated fish. This trend was the reverse of the pattern seen in the genes that did respond to thermal acclimation in muscle.

### The effects of T_2_ and T_3_ supplementation for responses sensitive to hypothyroid treatment

**Figure 4:**
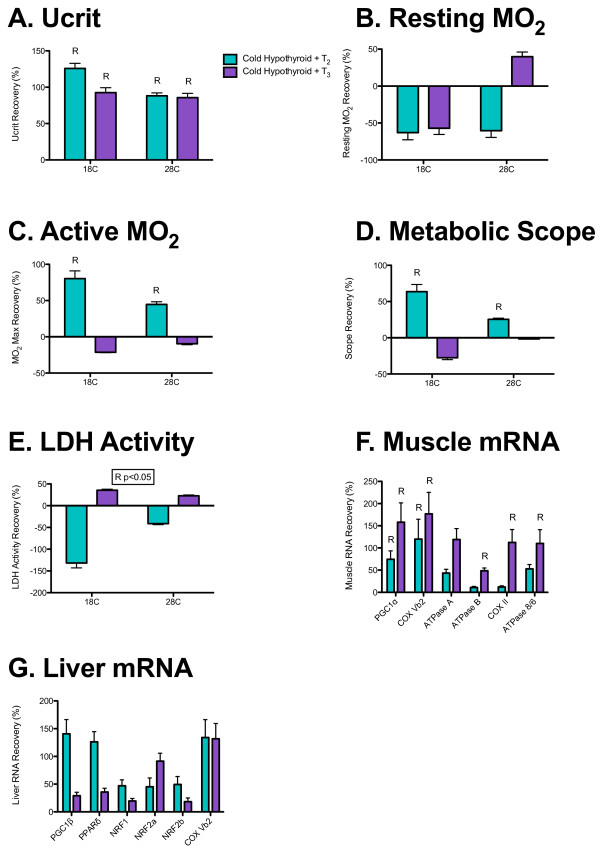
**Effects of T**_**2 **_**and T**_**3 **_**supplementation on thyroid hormone (TH)-sensitive measures in cold-acclimated fish.** Percentage recovery in (**A**) U_crit_, (**B**) resting metabolic rate, (**C**) active metabolic rate, (**D**) metabolic scope, (**E**) muscle LDH activity, (**F**) muscle mRNA transcript levels and **(G)** liver mRNA transcript levels in cold-acclimated hypothyroid fish supplemented with either T_2_ (teal) or T_3_ (purple); R, significant recovery with supplementation treatment; R *P*<0.05, significant effect of ANOVA where *post hoc* analyses were not significant.

Supplementation of hypothyroid fish with either T_2_ or T_3_ resulted in a significant recovery of sustained swimming performance in cold-acclimated fish at both the 18°C and 28°C test temperatures (Figure 4A; Table 6). There was no significant effect of T_2_ or T_3_ supplementation on resting metabolic rate, but T_2_ supplementation significantly restored active metabolic rate and metabolic scope at both test temperatures (Figure 4B,C; Table 4). There was a significant effect of T_2_ and T_3_ supplementation on muscle LDH activity, with T_2_ tending to decrease activity levels, and T_3_ increasing activity levels (Figure 4D; Table 6). In muscle, T_2_ supplementation resulted in a significant recovery of transcript levels for PGC1α and COX VB2, whereas T_3_ supplementation resulted in a significant recovery of transcript levels for PGC1α, COX VB2, ATPase B, COX II and ATPase 8/6 (Figure 4F; Table 7). In liver, T_2_ or T_3_ supplementation had no significant effect on the transcript levels of any of the genes previously shown to be sensitive to hypothyroid treatment (Figure 4G; Table 7).

**Table 6:** **Two-way ANOVA results comparing parameters between cold-acclimated normal thryoid, hypothyroid, and T**_**2**_**- or T**_**3**_**-supplemented fish**^**a**^

	**Thyroid status**			**Test temperature**			**Thyroid status × test temperature**		
	***F***	**d.f.**	***P***	***F***	**d.f.**	***P***	***F***	**d.f.**	***P***
U_crit_ ^b^	12.689	3, 65	0.001**	31.085	1, 65	0.001**	0.959	3, 65	0.417
RMR	1.466	3, 55	0.234	4.253	1, 55	0.044*	0.209	3, 55	0.890
AMR	3.535	3, 55	0.020*	11.032	1, 55	0.002**	0.050	3, 55	0.985
Scope	3.170	3, 55	0.006**	50.558	1, 55	0.005**	0.132	3, 55	0.899
LDH^c^	11.025	3, 64	0.001**	130.539	1, 64	0.001**	0.516	3, 64	0.673
COX^c^	1.421	3, 48	0.248	24.904	1, 48	0.001**	0.192	3, 55	0.902

Abbreviations: AMR, active metabolic rate; COX, cytochrome c oxidase; d.f., degrees of freedom; MS, metabolic scope; LDH, lactate dehydrogenase; RMR, resting metabolic rate.

^a^Citrate synthase was not measured because there was no significant effect of thyroid hormone in the cold-acclimated and warm-acclimated normal thyroid and hypothyroid fish.

^**b**^Swimming performance.

^**c**^Maximal activity.

*P<0.05.

**P<0.01.

**Table 7:** **One-way ANOVA results comparing muscle and liver mRNA levels between cold-acclimated normal thyroid, hypothyroid, and T**_**2**_**-supplemented or T**_**3**_**-supplemented fish**^**a**^

	**Muscle**			**Liver**		
**Gene**	***F***	**d.f.**	***P***	***F***	**d.f.**	***P***
PGC1α	10.233	3, 25	<0.001**	1.791	3, 39	0.165
PGC1β				2.202	3, 34	0.106
PPARδ				1.202	3, 38	0.322
NRF1				0.731	3, 29	0.542
NRF2a				1.272	3, 21	0.310
NRF2b				0.714	3, 23	0.554
COX Vb2	6.858	3, 28	0.001**	2.440	3, 21	0.093
ATPase A	2.422	3, 30	0.085			
ATPase B	3.380	3, 29	0.032*			
COX II	3.705	3, 23	0.026*			
ATP 8/6	5.530	3, 29	0.004**			

Abbreviations: ATPase A, F_0_F_1_-ATPase subunit A; ATPase B, F_0_F_1_-ATPase subunit B; ATPase 8/6, F_0_F_1_-ATPase subunits 8 and 6; COX II, Cytochrome c oxidase subunit 2; COX Vb2, Cytochrome c oxidase subunit 5b2; d.f. degrees of freedom; NRF1, Nuclear respiratory factor 1; NRF2a, Nuclear respiratory factor 2a; NRF2b, Nuclear respiratory factor 2b; PGC1α, Peroxisome proliferator-activated receptor γ coactivator 1-α; PGC1β, Peroxisome proliferator-activated receptor γ coactivator 1-β; PPARδ, Peroxisome proliferator-activated receptor δa and δb.

^a^Missing values represent genes that were not sensitive to TH in the cold-acclimated and warm-acclimated normal thyroid and hypothyroid fish, and therefore were not analyzed in cold-acclimated normal thyroid, hypothyroid, and T_2_-supplemented or T_3_-supplemented fish.

*P<0.05.

**P<0.01.

## Discussion

We have shown that TH regulates thermal acclimation of metabolism in an ectothermic vertebrate. Our principal novel findings were that 1) the actions of TH are temperature-specific, and 2) TH regulates thermal acclimation in ectotherms. We also showed that 3) T_2_ has a functional role in this thermal response, which, to our knowledge has not been shown in any other system. Thus, to our knowledge, this is the first time that an environmental factor as pervasive as temperature has been shown to determine not just the magnitude of a hormone-mediated response, but also the direction.

The traditional model for hormonal regulation is based on homeostatic control [24], by which the bioavailability of a hormone is adjusted to regulate its action. Opposing responses are typically mediated by antagonistic hormone pairs [37], but single hormones can also drive different responses depending upon the physiological context [24]. In the current study, we identified a novel signaling response, by which TH elicits a positive or negative response depending on the actual temperature and thermal history of the animal. TH has long been known to act in a tissue-specific manner [38,39], and it is possible that the same mechanisms that underlie its tissue specificity also underlie its temperature specificity. Phenotypic differences between tissues are primarily driven by differential patterns in gene expression defined during ontogeny [40,41], but gene-expression patterns, and therefore tissue phenotypes, are plastic, and can be adjusted in response to environmental factors such as temperature [1-3]. Thus, the thermal-acclimation response may change the tissue phenotype temporally to alter sensitivity to TH in a way that may parallel how different tissue types respond to TH.

The temperature specificity of TH action is evident at multiple levels of physiological organization, and mediates performance functions that determine fitness. We have shown that TH regulates energy metabolism and locomotor performance in response to chronic exposure to cold. This is the first time, to our knowledge, that a central regulator of thermal acclimation has been identified in an ectotherm, and provides a model that could explain vertebrate radiation during thermal-niche expansion. TH appears to have evolved as an environmental signaling molecule prior to vertebrate evolution [26-30,42,43]. In many invertebrates, TH suppresses larval structures, and promotes the growth and development of the juvenile rudiment [28]. Although many of these animals require exogenous THs ingested from food, others can synthesize THs or TH-like compounds endogenously [25,32,44-46]. It is interesting to note that in echinoderms, endogenously synthesized TH has been suggested to be a derived trait [25]. Growth and developmental rates are intrinsically linked to energy metabolism, and it is therefore likely that TH has always regulated these processes, at least in part by regulating metabolism. It is unknown whether the temperature specificity of TH is conserved in invertebrates, but it is conceivable that TH pathways evolved their sensitivity to temperature because both play such major roles in development [47-49]. If TH regulated metabolism to promote development at thermally challenging temperatures, then selection could favor this additional role. With an endogenous store of TH in the form of the thyroid gland, vertebrates could regulate these responses autonomously, and exploit novel thermal environments while maintaining important performance parameters such as locomotor capacity.

The properties that underlie the role of TH in thermal acclimation, temperature sensitivity, and metabolic control may have predisposed this hormone for a regulatory role in the evolution of endothermy. Of the response variables related to cold acclimation that we measured, most were highly sensitive to TH. The genes that were upregulated by TH during cold acclimation in zebrafish are homologous to those that control mammalian thermogenesis. In mammals, TH modulates the transcriptional regulation of metabolism by controlling expression levels of PGC1α [50], which plays a master role in coordinating the cross-genome expression of transcription factors involved in mitochondrial biogenesis and proteins that drive oxidative metabolism, including COX and F_0_F_1_-ATPase [51]. Our findings show that this same pathway underlies cold acclimation in an ectotherm. The most parsimonious explanation is that these pathways were conserved in early vertebrate ancestors; however, without similar analyses of other ectotherms, we cannot preclude the possibility that they were independently derived in both fish and mammals. PGC1α has also been shown to adjust skeletal muscle phenotype in ways that affect locomotion [52]. As is the case in mammals, PGC1α appears to be conserved as a target of TH in zebrafish, and probably also in other ectotherms. With the same pathways underlying both processes, the evolution of thermogenesis in mammals and birds [53-57] may have already been pre-programmed as a component of the cold-acclimation response in ectotherms.

T_3_ is often considered to be the only TH capable of genomic action because of its unique affinity for thyroid receptors [23]. However, T_2_ has recently been shown to stimulate metabolism in mammals and fish [23]. Our work supports the notion that T_2_ is also an important transcriptional regulator [58,59]. In many cases, T_2_ was just as effective as, if not more effective than, T_3_ at regulating the transcription of metabolic genes. Although T_2_ has poor affinity for thyroid receptors, it could exert its transcriptional control through cell surface receptors that are also known to respond to TH [5,18,60], or through reversible epigenetic modifications to histone complexes [61]. Importantly, the current study shows that T_2_ modulates performance parameters in the whole animal, which means that it is of ecological relevance and probably also of medical relevance. TH is associated with many modern lifestyle-induced conditions, and zebrafish have emerged as an important model for human endocrine diseases including obesity, diabetes, and metabolic syndromes [35,36]. The fact that T_2_ regulates metabolism and locomotor performance in a manner that is potentially very different from that of T_3_ means that the mechanistic basis of TH action is far broader than realized to date. A corollary is that thyroid-related diseases may be more complex, but also that there may be novel avenues for treatments that specifically target T_2_.

Several studies have measured changes in T_3_ and T_4_ plasma levels during thermal response in fish, but the combined results are ambiguous. In the current study we found a high level of variation in T_3_ levels in warm-acclimated euthyroid fish. There was also much variation in the TH levels of the fish receiving T_2_ and T_3_ supplement treatments, although all individuals measured had supplementation levels greater than or equal to their euthyroid counterparts. Overall, the muscle-specific concentrations of T_3_ and T_2_ in our study decreased drastically with cold acclimation, when their effects were most pronounced. However, TH concentrations alone do not indicate the bioavailability and/or bioactivity of the hormone within the target tissue. Instead, the action of TH is modulated by downstream regulators, which include plasma distributor proteins, TH transporters, deiodinase enzymes, intracellular reservoir proteins, target proteins (thyroid receptors, cell surface receptors, and specific enzymes), and transcriptional regulators [16-21,62]. The actions of TH may be more sensitive to changes in these downstream regulatory elements than to absolute free TH levels.

In many cases, the warm-acclimated zebrafish were far less sensitive to TH than cold-acclimated fish. This was especially evident in measures of locomotor performance. Decreased responsiveness to TH at warmer temperatures suggests that chronic increases in temperature, such as those brought about by global warming, will reduce the capacity of animals to adjust to environmental variation. Chronic warming could conceivably alter tissue phenotypes in ways that dampen, or reverse, TH-mediated response. This would interfere with TH as an environmental signaling molecule, and would compromise its crucial role in ectotherm growth and development. Importantly, many aquatic pollutants found worldwide, such as dioxins, bisphenol A, and phthalates, are thyroid-disrupting chemicals (TDCs) and bioaccumulate higher up the food chain [63]. Our findings indicate that the toxic effects of these chemicals may be temperature-specific, which is of crucial importance to the ecological influence of these pollutants. Together, climate change and rising levels of global pollution may amplify these risks. Warming temperatures are likely to result in higher concentrations, longer durations, and increased distributions of TDCs throughout the water column [64].

## Conclusions

Our finding that the effects of TH can depend upon the thermal history of an animal means that the toxicities of thyroid-disrupting pollutants, levels of which are increasing on a global basis, may vary with temperature. A corollary of this is that toxicological assessments of TDCs should consider the natural range of temperatures that a species experiences, or is predicted to experience in future. We show that TH is an important regulator of thermal acclimation in an ectotherm, which gives a new perspective on the evolution of thermal plasticity because it would be closely tied to the evolutionary history of the thyroid system. It is not known whether the role of TH in mediating thermal responses is conserved evolutionarily, or whether it is derived independently in mammals and earlier vertebrates. Based on our findings, we propose that the role of TH in thermal acclimation of fish predisposed it to evolve for regulatory control of thermogenesis. Decades of TH research have focused almost exclusively on T_3_, but the current study shows that that T_2_ also stimulates physiological activities that are of biological importance. Little is known about T_2_ signaling mechanisms, but the fact that it influences whole-animal performance independently from T_3_ suggests that it is important in both ecological and medical contexts.

## Methods

### Ethics statement

All experiments were carried out with the approval of the University of Sydney Animal Experimentation Ethics Committee (approval number L04/6-2010/2/5325).

### Animals and treatments

Zebrafish were purchased from commercial suppliers (Kim’s Aquatic World, Sydney, NSW, Australia, and Livefish, Bundaberg, QLD, Australia) and maintained at 23°C in 10-liter tanks of dechlorinated water at densities between 1.5 and 2 fish/l for at least 1 week before the start of the treatment regimens. Fish were fed *ad libitum* and maintained in a 14-hour light/10-hour dark photoperiod.

Fish were split into two temperature treatments, a cold acclimation group kept at 18°C, and a warm acclimation group at 28°C, and held at these temperatures (±0.5°C) for 3 weeks. Within these acclimation groups, fish were separated into a normal thyroid group and a hypothyroid treatment group. Within the cold-acclimated hypothyroid group, fish were further divided into three treatment groups to be given daily supplements of T_3_ (3,5,3^′^-triiodothyronine; Sigma), T_2_ (3,5-diiodothyronine; Sigma), or the ethanol vehicle. There were five replicate tanks per treatment.

We induced hypothyroidism by maintaining the tank water with 0.3 mmol/l propylthiouracil (PTU; Sigma, Australia), which blocks the production of T_4_ at the thyroid gland [23], dissolved in a DMSO vehicle. Every 4 days, 80% of the tank water was changed, and the PTU was added again to return the concentration to 0.3 mmol/l. The hypothyroid groups were also treated with 5 μmol/l iopanoic acid (Thermofisher Scientific Inc., Australia) daily to inhibit deiodinase activity, thereby preventing the peripheral metabolism of TH. Tanks holding the normal thyroid groups were maintained with proportionate amounts of the respective vehicles (0.05% DMSO and 0.025% ethanol).

### Sustained swim performance

Sustained swim performance (critical sustained swim speed, U_crit_[65]) was measured in a flume consisting of a clear Perspex tube 150 mm in length and 26 mm in diameter, tightly fitted into the single exit of a Y-shaped rubber connector (total length 0.15 meters). Two 12 V inline submersible pumps (iL500; Rule, Miami, FL, USA) were fitted into the other openings of the Y connector. A plastic grid separated the flume from the two pumps, and bundles of hollow straws were positioned at each end of the flume to promote laminar flow. The flume and pumps were submerged in a plastic tank (645 × 423 × 276 mm). We used a variable DC power source (MP3090; Powertech, Osborne Park, WA, Australia) to adjust the flow speed, which was calibrated using a flow meter (FP101; Global Water, Gold River, CA, USA). U_crit_ was determined in accordance with published protocols [65,66], with a time interval between speed increments of 600 seconds, a speed increment of 0.06 meters and an initial flow rate of 0.2 m/s. Fish were allowed 15 minutes to equilibrate to the flume conditions before swim trials were begun. Animals were swum until fatigued, which was defined as the time when fish could no longer hold their position in the water column [65]. Each fish was swum at 18°C and 28°C in random order, with at least 24 hours between swim trials. U_crit_ is reported as body length (BL) per second (s).

### Metabolic scope

Resting and maximum rates of O_2_ consumption were analyzed with a fiber optic oxygen sensor (Microx 1/FIBOX; PreSens, Regensburg, Germany) in custom-made metabolic chambers (circular sealable plastic containers of 90 ml volume) at 18°C and 28°C. For resting rates of O_2_ consumption, fish were placed inside dark sealed chambers continuously supplied with oxygenated water via input tubes for at least 60 minutes before trials began, to allow them to equilibrate. Preliminary measures of metabolic rate over 24 hours in the same setup showed that the zebrafish reached resting status within 60 minutes. After the equilibration period, the flow of oxygenated water was stopped, and the tubes servicing each chamber were sealed via plastic valves to ensure a closed system. An initial [O_2_] reading was taken, followed by a final [O_2_] reading after 20 minutes. The total O_2_ depletion over this period was used to calculate the mass-specific resting metabolic rate. To measure the maximum rate of O_2_ consumption, a magnetic stir bar was added into the same chamber and partitioned from the bottom portion with a wire mesh. The chamber was placed on a magnetic stirring plate and stirring intensity was used to manipulate flow speed. A plastic column was placed vertically in the center of the chamber to reduce turbulence. To ensure active status was reached, fish were swum for 2 minutes before the initial [O_2_] was measured. Final [O_2_] was measured at 7 minutes. The total O_2_ depletion over 5 minutes was used to calculate mass-specific active metabolic rate. Fish were monitored for the full 7-minute trial, and water flow was adjusted so that the fish were swum at maximum capacity, which was defined as when the fish visibly struggled to hold their position in the water column. Oxygen concentration was also measured in empty metabolic chambers at both test temperatures to control for alternative sources of oxygen depletion. Rate of oxygen consumption was calculated using the formula

$$\left(\left(C_{2}–C_{1}\right)\times V\right)/\left(M\times T\right),$$

where C_1_ is the initial concentration of O_2_, C_2_ is the final concentration of O_2_, V is the volume of the chamber, M is the mass of the fish and T is the trial time. Metabolic scope was calculated as the difference between active and resting metabolic rates.

### Thyroid hormone quantification

Unfortunately, the limits of detection did not allow us to analyze the fish liver tissue because of its small size. Levels of T_3_ and T_2_ (Table 3, Table 4) were quantified in muscle tissue as described previously [67,68], and expressed as means, and as ranges of the mean in cases where the TH metabolite was detected in amounts below the limits of quantification.

### Enzyme assays

We measured the activities of LDH, CS and COX to assess how acclimation temperature and thyroid status affect maximal rates of metabolic enzymes. We chose these enzymes because they are important components of glycolytic and oxidative metabolism [69,70]. Tail muscle was collected, transferred into liquid nitrogen, and stored at -80°C for later analysis. Enzyme activities were determined in accordance with published protocols [71].

### mRNA concentrations

Upon completion of treatments, the zebrafish were euthanized by immersion in a buffered MS222 (tricaine methane sulfonate) solution (0.4 g/l MS222 + 0.8 g/l Na_2_HCO_3_). Liver and muscle were dissected and stored in RNAlater (Ambion, USA) at -20°C. RNA was extracted from samples (TRIreagent; Molecular Research Center, Cincinnati, OH, USA), in accordance with the manufacturer’s instructions. RNA concentration and quality were verified using a spectrophotometer (NanoDrop Technologies, Australia) and a microfluidics-based platform (Bioanalyzer 2100; Agilent Tecnologies, Australia) when necessary. An aliquot (2 μg) of total RNA from each sample was treated with DNAse I (Sigma-Aldrich) and reverse-transcribed using RNAse HMMLV reverse transcriptase (Bioscript; Bioline, Australia) and random hexamer primers (Bioline).

Quantitative reverse transcriptase (qRT)-PCR was performed on a qRT-PCR machine ( 7500; Applied Biosystems, Foster City, CA, USA) in accordance with published protocols [66]. Primers for important transcriptional regulators of metabolic enzymes, peroxisome proliferator-activated receptor γ coactivators 1-α and 1-β (PGC1α and PGC1β), peroxisome proliferator-activated receptors δa and δb (PPARδ), nuclear respiratory factors 1, 2a and 2b (NRF1, NRF2a and NRF2b), and subunits of metabolic enzymes, cytochrome c oxidase subunits VB2 and II (COX VB2 and COX II), and F_0_F_1_-ATPase subunits A, B, 8 and 6 (ATPase A, ATPase B and ATPase 8/6) were adopted from published works, or designed from their respective Genbank sequences (see Additional file 2: Table S2). The gene transcripts were chosen because they encode enzymes, enzyme subunits, transcription factors, and coactivators that are important regulators of oxidative phosphorylation [3,70,72]. All qPCRs were run in 96-well optical plates (BIOplastics, Landgraaf, the Netherlands). Real-time PCR reactions contained 1× SensiMix SYBR (Bioline, Australia), 4.5 mmol/l MgCl_2_, 50 to 900 nmol/l of each primer and approximately 100 ng cDNA. The cycle consisted of 95°C for 7 minutes, followed by 40 cycles of 95°C for 20 seconds and 58°C for 1 minute. Dissociation-curve analysis was performed after the amplification step to verify the presence of only a single PCR product. Transcript expression levels of the 11 target genes in each treatment group were normalized to elongation factor 1 (EF1)-α in accordance with recent recommendations for zebrafish housekeeping genes [73], and were expressed relative to the warm-acclimated normal thyroid treatment for the warm/cold hypothyroid experiment, and to the cold-acclimated normal thyroid treatment for the T_3_/T_2_ supplementation experiment.

### Statistical analyses

Data are presented as means ± standard error of the mean (SEM) Trifactorial datasets (acclimation temperature × thyroid status × test temperature) and bifactorial datasets for mRNA analysis (acclimation temperature × thyroid status) were analyzed by permutational multivariate analysis of variance (PERMANOVA) using the Primer 6 and PERMANOVA+ packages (PRIMER-E Ltd, Plymouth, Cornwall UK). In the case of interactions involving acclimation temperature and test temperature, *a priori* planned contrasts were conducted in PERMANOVA to determine the main effects of acclimation temperature in the normal thyroid treatments, and of thyroid status in the cold and warm acclimation treatments. The remaining bifactorial datasets (thyroid status × test temperature) and single-factor datasets (thyroid status) were analyzed by ANOVA followed by Tukey *post hoc* tests using PASW Statistics 18. Where ANOVA was used, all data were tested for normality and homogeneity of variance using Levene’s test. If a dataset tested significant for Levene’s test, it was transformed (log_10_, sqrt, *x*^2^). Significance was considered as *P*<0.05.

We used the truncated product method [74] to assess the effect of multiple comparisons on the validity of *P* values. Briefly, the truncated product method considers the distribution of *P* values from multiple hypothesis tests to provide a table-wide *P* value for the overall hypothesis that *P* values were not skewed, leading to type 1 errors. Multiple hypothesis testing did not bias the statistical results presented here (*P* <0.0001).

### Data presentation

The effects of acclimation treatment are presented as absolute values for U_crit_, metabolic rate, metabolic scope, and maximal enzyme activity, and as relative values for mRNA transcript levels. For hypothyroid treatments, the data are presented as percentage change from the normal thyroid treatments:

(normal thyroid treatment value – hypothyroid treatment value)×100.

For T_2_ and T_3_ supplementation treatments, the data are presented as percentage recovery from hypothyroid treatment

$$\begin{array}{c} \text{((supplemented treatment value – hypothyroid treatment value)/}\\ \text{ (normal thyroid treatment value – hypothyroid treatment value))}\times \text{100.} \end{array}$$

## Abbreviations

ANOVA: analysis of variance; ATPase A: F_0_F_1_-ATPase subunit A; ATPase B: F_0_F_1_-ATPase subunit B; ATPase 8/6: F_0_F_1_-ATPase subunits 8 and 6; COX: Cytochrome c oxidase; COX II: Cytochrome c oxidase subunit 2; COX Vb2: Cytochrome c oxidase subunit 5b2; CS: Citrate synthase; d.f.: degrees of freedom; DMSO: dimethyl sulfoxide; LDH: Lactate dehydrogenase; NRF1: Nuclear respiratory factor 1; NRF2a: Nuclear respiratory factor 2a; NRF2b: Nuclear respiratory factor 2b; nTRE: Negative thyroid response element; PERMANOVA: permutational multivariate analysis of variance; PGC1α: Peroxisome proliferator-activated receptor γ coactivator 1-α; PGC1β: Peroxisome proliferator-activated receptor γ coactivator 1-β; PPARδ: Peroxisome proliferator-activated receptor δa and δb; T2: 3,5-diiodothyronine; T3: 3,5,3^′^-triiodothyronine; TH: Thyroid hormone; TDC: Thyroid-disrupting chemical; TRE: Thyroid response element

## Competing interest

The authors declare no competing interest.

## Authors’ contributions

AGL and FS conceived the project and designed the experiments; TS and KK developed the analytical protocols; AGL, TS and KK performed the experiments; AGL analyzed the data; and AGL and FS wrote the manuscript. All authors read and approved the final manuscript.

## Supplementary Material

Additional file 1: Table S1 Pairwise planned comparison results comparing the effects of acclimation temperature between cold control (CC) and warm control (WC) groups, and of hypothyroidism between CC and cold hypothyroid (CH) treatments and WC and warm hypothyroid (WH) treatments. *P*_MC_, Monte Carlo *P*-value; t, *t*-value; d.f., degrees of freedom; m-, muscle RNA; l-, liver RNA. Click here for file

Additional file 2: Table S2 List of primers used in this study with original source (published article or Genbank accession number for which primers were designed). Click here for file
