# Supplementary material for: Thyroid hormone actions are temperature-specific and regulate thermal acclimation in zebrafish (Danio rerio)
Source: BMC Biol. 2013 Mar 26;11:26. doi: 10.1186/1741-7007-11-26 (PMC3633057; doi:10.1186/1741-7007-11-26)
Supplement: Additional file 1: Table S1 — Pairwise planned comparison results comparing the effects of acclimation temperature between cold control (CC) and warm control (WC) groups, and of hypothyroidism between CC and cold hypothyroid (CH) treatments and WC and warm hypothyroid (WH) treatments. PMC, Monte Carlo P-value; t, t-value; d.f., degrees of freedom; m-, muscle RNA; l-, liver RNA. [file 1741-7007-11-26-S1.docx]

**Table S1.** Pairwise planned comparison results comparing the effects of acclimation temperature between cold control (CC) and warm control (WC) groups, and of hypothyroidism between cold control and cold hypothyroid treatments (CH) and warm control and warm hypothyroid (WH) treatments; Monte Carlo p-value (p_MC_), t-value (t), degrees of freedom (df), muscle RNA (m-) and liver RNA (l-).

|  | **Groups** | **P_MC_** | **t** | **df** |
| --- | --- | --- | --- | --- |
| **Ucrit** | CCxWC** | 0.001 | 4.729 | 33 |
|  | CCxCH* | 0.001 | 4.914 | 33 |
|  | WCxWH | 0.38 | 0.908 | 33 |
| **LDH activity** | CCxWC* | 0.037 | 2.060 | 36 |
|  | CCxCH** | 0.001 | 4.038 | 36 |
|  | WCxWH | 0.423 | 0.793 | 36 |
| **COX activity** | CCxWC | 0.664 | 0.451 | 28 |
|  | CCxCH | 0.732 | 0.411 | 28 |
|  | WCxWH* | 0.012 | 2.481 | 28 |
| **m-PGC1a** | CCxWC** | 0.005 | 2.759 | 18 |
|  | CCxCH** | 0.002 | 3.880 | 15 |
|  | WCxWH | 0.892 | 0.361 | 15 |
| **m-COXVB2** | CCxWC** | 0.002 | 2.607 | 17 |
|  | CCxCH** | 0.002 | 3.396 | 15 |
|  | WCxWH | 0.281 | 1.135 | 15 |
| **m-ATPaseA** | CCxWC** | 0.004 | 2.717 | 17 |
|  | CCxCH* | 0.024 | 2.400 | 12 |
|  | WCxWH | 0.050 | 0.804 | 12 |
| **m-ATPaseB** | CCxWC** | 0.004 | 2.766 | 10 |
|  | CCxCH* | 0.027 | 2.263 | 15 |
|  | WCxWH | 0.123 | 1.504 | 15 |
| **l-PGC1B** | CCxWC | 0.092 | 1.654 | 15 |
|  | CCxCH* | 0.049 | 1.993 | 17 |
|  | WCxWH* | 0.046 | 2.094 | 17 |
| **l-PPARd** | CCxWC | 0.123 | 1.531 | 15 |
|  | CCxCH* | 0.050 | 2.065 | 19 |
|  | WCxWH | 0.068 | 1.812 | 12 |
| **l-NRF1** | CCxWC** | 0.012 | 2.536 | 13 |
|  | CCxCH* | 0.041 | 2.104 | 14 |
|  | WCxWH | 0.383 | 0.934 | 12 |
| **l-NRF2a** | CCxWC | 0.510 | 0.774 | 11 |
|  | CCxCH* | 0.019 | 2.669 | 10 |
|  | WCxWH | 0.128 | 1.553 | 12 |
| **l-NRF2b** | CCxWC | 0.272 | 1.121 | 13 |
|  | CCxCH* | 0.015 | 2.705 | 12 |
|  | WCxWH | 0.096 | 1.717 | 12 |
| **l-COXII** | CCxWC | 0.093 | 0.230 | 13 |
|  | CCxCH | 0.083 | 0.792 | 12 |
|  | WCxWH* | 0.043 | 2.027 | 10 |
| **l-ATP8/6** | CCxWC | 0.916 | 0.230 | 11 |
|  | CCxCH | 0.159 | 1.466 | 10 |
|  | WCxWH | 0.155 | 1.494 | 11 |

***p<0.05**

****p<0.01**
