# Supplementary material for: Thyroid hormone actions are temperature-specific and regulate thermal acclimation in zebrafish (Danio rerio)
Source: BMC Biol. 2013 Mar 26;11:26. doi: 10.1186/1741-7007-11-26 (PMC3633057; doi:10.1186/1741-7007-11-26)
Supplement: Additional file 2: Table S2 — List of primers used in this study with original source (published article or Genbank accession number for which primers were designed). [file 1741-7007-11-26-S2.docx]

**Supporting Information**

**Table S2.** List of primers used in this study with original source (published article or Genbank accession number for which primers were designed).

|  | **Forward Sequence** | **Reverse Sequence** | **Source** |
| --- | --- | --- | --- |
| **PGC1α** | 5’ttggatgcttcattgccata3’ | 5’ctgcctgagcttgacctttc3’ | Seebacher and Walter 2012 |
| **PGC1β** | 5’aagccagtatggggaagagg3’ | 5’ccagcgctgtactgtatgga3’ | Seebacher and Walter 2012 |
| **PPARδ** | 5’atcgtccwggsctsatgaac3’ | 5’aggtcsgccakcttctgc3’ | Seebacher and Walter 2012 |
| **NRF1** | 5’aggccctgaggactatcgtt3’ | 5’gctccagtgccaacctgtat3’ | McClelland et al. 2006 |
| **NRF2a** | 5’tggagattgtgaagccagtg3’ | 5’ttcagtcgaacctgctcctt3’ | BC165340.1 |
| **NRF2b** | 5’gcggatgtgaacgctaaag3’ | 5’gtccagagcgttcttgcag3’ | NM_001077450.1 |
| **COX Vb2** | 5’gcccagcatcaataacaaacg3’ | 5’aagcgctgtacatggcagaa3’ | NM_001045357.1 |
| **ATPase A** | 5’gctttcgctcagtttggttc3’ | 5’aaggccttctcgaatttggt3’ | NM_001077355 |
| **ATPase B** | 5’tcagaggtgtctgcccttct3’ | 5’agtcatcagggggcacatag3’ | NM_001089565.1 |
| **COX II** | 5’ttccggccatcattcttatt3’ | 5’gtgtgaggtcttgggttggt3’ | AC024175.3 |
| **ATP 8/6** | 5’aacccccatcccattaattc3’ | 5’aacccccatcccattaattc3’ | AC024175.3 |
| **ELF1α** | 5’cttctcaggctgactgtgc3’ | 5’ccgctagcattaccctcc3’ | McCurley and Callard, 2008 |
